# Excavatoids O and P, New 12-Hydroxybriaranes from the Octocoral *Briareum excavatum*

**DOI:** 10.3390/md8102639

**Published:** 2010-10-12

**Authors:** Ping-Jyun Sung, Gung-Ying Li, Yin-Di Su, Mei-Ru Lin, Yu-Chia Chang, Ting-Hsuan Kung, Chan-Shing Lin, Yung-Husan Chen, Jui-Hsin Su, Mei-Chin Lu, Jimmy Kuo, Ching-Feng Weng, Tsong-Long Hwang

**Affiliations:** 1 Graduate Institute of Marine Biotechnology, National Dong Hwa University, Pingtung 944, Taiwan; E-Mails: johnny9210014@hotmail.com (G.-Y.L.); x2219@nmmba.gov.tw (J.-H.S.); jinx6609@yahoo.com.tw (M.-C.L.); jimmy@nmmba.gov.tw (J.K.); 2 National Museum of Marine Biology & Aquarium, Pingtung 944, Taiwan; E-Mails: gobetter04@yahoo.com.tw (Y.-D.S.); linmeiru@hotmail.com (M.-R.L.); jay0404@gmail.com (Y.-C.C.); sevenapril@nmmba.gov.tw (T.-H.K.); tony_chen72001@yahoo.com.tw (Y.-H.C.); 3 Department of Life Science and the Institute of Biotechnology, National Dong Hwa University, Hualien 974, Taiwan; E-Mail: cfweng@mail.ndhu.edu.tw (C.-F.W.); 4 Department of Marine Biotechnology and Resources, National Sun Yat-sen University, Kaohsiung 804, Taiwan; E-Mail: shinlin@mail.nsysu.edu.tw (C.-S.L.); 5 Asia-Pacific Ocean Research Center, National Sun Yat-sen University, Kaohsiung 804, Taiwan; 6 Doctoral Degree Program in Marine Biotechnology, National Sun Yat-sen University, Kaohsiung 804, Taiwan; 7 Graduate Institute of Natural Products, Chang Gung University, Taoyuan 333, Taiwan; E-Mail: htl@mail.cgu.edu.tw (T.-L.H.)

**Keywords:** excavatoid, briarane, octocoral, Briareum excavatum

## Abstract

Two new 12-hydroxybriarane diterpenoids, designated as excavatoids O (**1**) and P (**2**), were isolated from the octocoral *Briareum excavatum*. The structures of briaranes **1** and **2** were established on the basis of extensive spectral data analysis. Excavatoid P (**2**) is the first metabolite which possesses a 6*β* -chlorine atom in briarane analogues.

## 1. Introduction

**Scheme 1:**
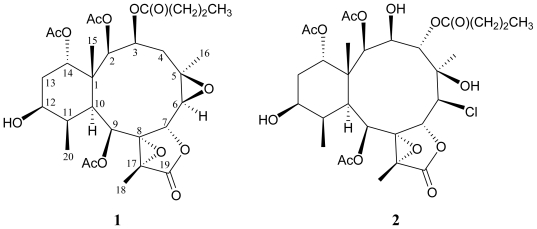
The Structures of Excavatoids O (**1**) and P (**2**).

In our research on the chemical constituents of the marine invertebrates collected in Taiwan waters, a series of briarane-type diterpenoid derivatives had been isolated from various octocorals belonging to the genus *Briareum* (family Briareidae), *Ellisella*, and *Junceella* (family Ellisellidae), and the compounds of this type were proven to possess various interesting bioactivities [1–3]. Recently, our further chemical examination of *Briareum excavatum* has resulted in the isolation of two new highly oxidized briarane-type diterpenoids, excavatoids O (**1**) and P (**2**) (Scheme 1). The structures of compounds **1** and **2** were established by spectroscopic methods.

## 2. Results and Discussion

Excavatoid O (**1**) was obtained as a white powder and had a molecular formula C_30_H_42_O_13_, as determined by HRESIMS (C_30_H_42_O_13_ + Na, *m/z* found 633.2519; calculated 633.2523) indicating 10 degrees of unsaturation. The presence of hydroxy, lactone, and ester groups in **1** were evidenced by the IR absorptions at 3512, 1793, and 1741 cm^−1^. It was found that the ^1^H and ^13^C spectra of **1** in CDCl_3_ revealed mostly broad peaks when measured at 25 °C. In order to make more reliable assignments of NMR signals of the stabilized conformers, the ^1^H and ^13^C NMR spectra of **1** were measured at 0 °C in CDCl_3_. In the ^13^C spectrum of **1**, five ester carbonyl resonances appeared at *δ*_C_ 173.6, 170.8, 170.1, 169.5, and 169.3 (5 × s) (Table 1). In the above carbonyl carbons, three were identified as acetate carbonyls by the presence of three methyl resonances in the ^1^H NMR spectrum at *δ*_H_ 2.18, 2.12, and 1.96 (each 3H × s) and one was identified as *n*-butyrate carbonyl by the presence of seven contiguous protons at *δ*_H_ 0.95 (3H, t, *J* = 7.2 Hz), 1.64 (2H, m), and 2.23 (2H, m) (Table 1). On the basis of the unsaturation data overall, **1** was concluded to be a briarane diterpenoid molecule possessing five rings. A tetra-substituted epoxide containing a methyl substituent was elucidated from the signals of two oxygenated quaternary carbons at *δ*_C_ 72.6 (s, C-8) and 63.3 (s, C-17); and further confirmed by the proton signal of a methyl singlet at *δ*_H_ 1.57 (3H, s, H_3_-18). In addition, a tri-substituted epoxide containing a methyl substituent was deduced from the signals of an oxymethine (*δ*_H_ 3.11, 1H, d, *J* = 8.8 Hz, H-6; *δ*_C_ 63.0, d, C-6), a quaternary oxygen-bearing carbon (*δ*_C_ 62.1, s, C-5), and a methyl singlet at δ_H_ 1.35 (3H, s, H_3_-16).

From the ^1^H-^1^H COSY experiment of **1** (Table 2), it was possible to establish the separate spin systems that map out the proton sequences from H-2/H-3/H_2_-4, H-6/H-7, and H-9/H-10. These data, together with the HMBC correlations between H-2/C-1, -4; H-3/C-4; H_2_-4/C-3, -5, -6; H-7/C-5, -6; H-9/C-1, -7, -8, -10; and H-10/C-1, -2, established the connectivity from C-1 to C-10 in the 10-membered ring (Table 2). The methyl group at C-5 was confirmed by the HMBC correlations between H_3_-16/C-4, -5, -6. The methylcyclohexane ring, which is fused to the 10-membered ring at C-1 and C-10, was elucidated by the ^1^H-^1^H COSY correlations between H-10/H-11/H-12/H_2_-13/H-14 and H-11/H_3_-20 and by the HMBC correlations between H-2/C-14; H-9/C-11; H-10/C-11, -12, -14; H-11/C-10, -20; H_2_-13/C-1; H-14/C-1, -2; and H_3_-20/C-10, -11, -12. The ring junction C-15 methyl group was positioned at C-1 from the HMBC correlations between H-2/C-15; and H_3_-15/C-1, -2, -10, -14. In addition, the HMBC correlations also revealed that three acetates should be attached at C-2, C-9, and C-14, respectively. The remaining *n*-butyrate ester and hydroxy groups were positioned at C-3 and C-12 as indicated by analysis of ^1^H-^1^H COSY correlations and characteristic NMR signals analysis (*δ*_H_ 5.13, 1H, br s, H-3; *δ*_C_ 69.9, d, C-3; *δ*_H_ 3.96, 1H, br s, H-12; *δ*_C_ 69.3, d, C-12). These data, together with the HMBC correlations between H-7/C-17, -19 and H_3_-18/C-8, -17, -19, were used to establish the molecular framework of **1**.

**Figure 1:**
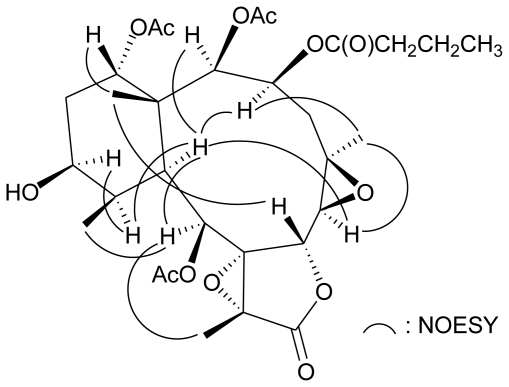
Selective NOESY correlations of **1**.

Based on previous studies, all naturally occurring briarane-type diterpenoids have the C-15 methyl group as *trans* to H-10, and these two groups are assigned as *β* - and *α*-oriented, respectively, as shown in most briarane derivatives [1–3]. The relative stereochemistry of **1** was established from a NOESY experiment (Figure 1). In the NOESY experiment of **1**, the correlations of H-10 with H-2, H-3, H-6, H-9, and H-11; and H-11 correlated with H-12, indicated that these protons are situated on the same face and were assigned as *α* protons since the C-15 methyl is the *β* -substituent at C-1. H-14 was found to exhibit a correlation with H_3_-15, revealing the *β* -orientation of this proton. The correlations between H_3_-16 and H-3, H-6, reflected the *α*-orientation of H_3_-16. H-7 correlated with H_3_-15, indicating this proton should be *β* -oriented. Furthermore, H_3_-18 showed a correlation with H-9. By detailed analysis of molecular models, H_3_-18 was found to be reasonably close to H-9 when H_3_-18 was placed on the *β* face in the *γ*-lactone moiety. Based on the above findings, the structure of **1** was elucidated unambiguously.

The molecular formula of excavatoid P (**2**) was determined as C_30_H_43_ClO_14_ by its HRESIMS (*m/z* 685.2235, calculated for C_30_H_43_ClO_14_ + Na, 685.2239). The IR spectrum showed bands at 3472, 1784, and 1734 cm^−1^, consistent with the presence of hydroxy, *γ*-lactone, and ester groups in **2**. From the ^13^C NMR data of **2** (Table 1), five carbonyl resonances appeared at *δ*_C_ 173.9, 172.0, 170.4, 170.3, and 170.0 (5 × s), confirming the presence of a *γ*-lactone and four esters in **2**; three acetyl methyls (*δ*_H_ 2.43, 2.17, 2.03, each 3H × s) and an *n*-butyryl group (*δ*_H_ 0.98, 3H, t, *J* = 7.6 Hz; 1.66, 2H, m; 2.33, 2H, t, *J* = 7.6 Hz) were also observed. According to the above data, briarane **2** was found to be a tetracyclic compound with a *γ*-lactone, as no other unsaturated functional group could be found.

^1^H NMR coupling information in the ^1^H-^1^H COSY spectrum of **2** enabled identification of the C-2/-3/-4, C-6/-7, C-9/-10/-11/-12/-13/-14, and C-11/-20 units (Table 2), which were assembled with the assistance of an HMBC experiment (Table 2). The HMBC correlations between protons and quaternary carbons, such as H-2, H-3, H-10, H-11, H_3_-15/C-1; H-3, H-4, H-6, H-7, H_3_-16, OH-5/C-5; H-6, H-9, H-10, H_3_-18/C-8; H-9, H_3_-18/C-17; and H-7, H_3_-18/C-19, permitted elucidation of the carbon skeleton. A methyl at C-5 was established by the HMBC correlations between H_3_-16/C-4, -5, -6 and H-4, H-6, OH-5/C-16. The ring junction C-15 methyl group was positioned at C-1 from the HMBC correlations between H_3_-15/C-1, -2, -10, -14; and H-10/C-15. The acetate esters at C-2 and C-9 were established by correlations between H-2 (*δ*_H_ 4.62), H-9 (*δ*_H_ 5.36) and the acetate carbonyls observed in the HMBC spectrum of **2**. The *n*-butyrate ester positioned at C-4 was confirmed from the HMBC correlation between H-4 (*δ*_H_ 5.82) and the carbonyl carbon (*δ*_C_ 173.9) of *n*-butyrate ester. Thus, the remaining acetoxy group was positioned at C-14 as indicated by analysis of ^1^H-^1^H COSY correlations and characteristic NMR signals (*δ*_H_ 4.84, 1H, br s, H-14; *δ*_C_ 80.8, d, C-14). The presence of hydroxy groups at C-3 and C-12 was deduced from the ^1^H-^1^H COSY correlations between the hydroxy protons (*δ*_H_ 3.18, OH-3 and *δ*_H_ 2.17, OH-12) and H-3 (*δ*_H_ 5.07) and H-12 (*δ*_H_ 4.13), respectively. The C-5 hydroxy group was also confirmed by the HMBC correlations between the hydroxy proton (*δ*_H_ 2.36, OH-5) and C-4, -5, -16. Thus, the remaining chlorine atom in **2** should be attached C-6 by the ^1^H-^1^H COSY correlation between H-6 (*δ*_H_ 4.29) and H-7 (*δ*_H_ 5.26) and further supported by the HMBC correlations between H-6/C-4, -5, -7, -8, -16 and H-7, H_3_-16/C-6.

**Figure 2:**
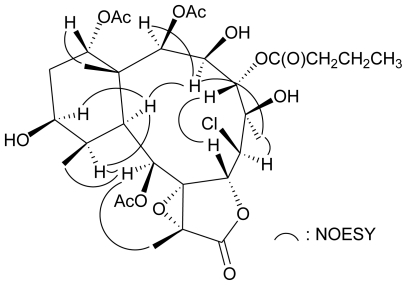
Selective NOESY correlations of **2**.

The relative configuration of **2** was elucidated from the interactions observed in a NOESY experiment and from vicinal proton coupling constant analysis. In the NOESY experiment of **2** (Figure 2), the correlations of H-10 with H-3, H-11, and H-12, indicated that these protons were situated on the same face and were assigned as *α* protons since the C-15 and C-20 methyls are *β* -substituents at C-1 and C-11, respectively. H-2 exhibited an interaction with H-3, and no coupling was found between H-2 and H-3, indicating that the dihedral angle between H-2/H-3 is approximately 90° and the acetoxy group at C-2 should be *β* -oriented. H-14 was found to exhibit a response with H_3_-15, showing that H-14 has a *β* -orientation. H-9 was found to show responses with H-11, H_3_-18, and H_3_-20. From modeling analysis, H-9 was found to be close to H-11, H_3_-18, and H_3_-20 when H-9 was *α*-oriented. Moreover, H_3_-16 exhibited correlations with H-3 and H-6, and no coupling constant was detected between H-6 and H-7, suggesting the *α*-orientation of H_3_-16 and H-6; and *β*-orientation of H-7. H-7 exhibited a correlation with H-4, and no coupling was found between H-3 and H-4, indicating that the dihedral angle between H-3 and H-4 is also approximately 90° and the *n*-butyrate ester group at C-4 was *α*-oriented. On the basis of the above results, the structure of **2** was elucidated. To the best of our knowledge, briarane **2** is the first briarane which possesses a 6*β* -chlorine atom.

In the biological activity testing, briaranes **1** and **2** displayed 16.9 and 16.1% inhibitory effects on elastase release by human neutrophils at 10 μg/mL, resepectively [4].

## 3. Experimental

### 3.1. General Experimental Procedures

Melting points were determined on a FARGO apparatus and were uncorrected. Optical rotation values were measured with a JASCO P-1010 digital polarimeter at 25 °C. Infrared spectra were obtained on a VARIAN DIGLAB FTS 1000 FT-IR spectrometer. The NMR spectra were recorded on a VARIAN MERCURY PLUS 400 FT-NMR at 400 MHz for ^1^H and 100 MHz for ^13^C, in CDCl_3_, at 25 or 0 °C, respectively. Proton chemical shifts were referenced to the residual CHCl_3_ signal (*δ*_H_ 7.26 ppm). ^13^C NMR spectra were referenced to the center peak of CDCl_3_ at *δ*_C_ 77.1 ppm. ESIMS and HRESIMS data were recorded on a BRUKER APEX II mass spectrometer. Column chromatography was performed on silica gel (230–400 mesh, Merck, Darmstadt, Germany). TLC was carried out on precoated Kieselgel 60 F_254_ (0.25 mm, Merck) and spots were visualized by spraying with 10% H_2_SO_4_ solution followed by heating. HPLC was performed using a system comprised of a HITACHI L-7100 pump, a HITACHI photodiode array detector L-7455, and a RHEODYNE 7725 injection port. A normal phase column (Hibar 250 × 10 mm, Merck, silica gel 60, 5 μm,) was used for HPLC.

### 3.2. Animal Material

Specimens of the octocoral *Briareum excavatum* were collected and transplanted in 0.6-ton cultivating tanks located in the NMMBA, Taiwan, in December 2003. This organism was identified by comparison with previous descriptions [5–7]. A voucher specimen was deposited in the National Museum of Marine Biology and Aquarium, Taiwan.

### 3.3. Extraction and Isolation

The organism (wet weight 1.0 kg) was collected and freeze-dried. The freeze-dried material was minced and extracted with EtOAc. The extract was separated by silica gel column chromatography, using hexane and hexane/EtOAc mixtures of increased polarity to yield 12 fractions. Fraction 3 was separated by normal phase HPLC, using a mixture of dichloromethane and acetone to afford briarane **2** (0.9 mg, 9/1). Fraction 2 was separated by normal phase HPLC, using a mixture of hexane and EtOAc to afford briarane **1** (13.2 mg, 1/1).

Excavatoid O (**1**): white powder; mp 137–138 °C; [*α*]_D_^25^ − 39 (*c* 0.4, CHCl_3_); IR (neat) *ν*_max_ 3512, 1793, 1741 cm^−1^; ^13^C NMR (CDCl_3_, 100 MHz) and ^1^H NMR (CDCl_3_, 400 MHz) data, see Table 1; ESIMS *m/z* 633 (M + Na)^+^; HRESIMS *m/z* 633.2519 (Calcd for C_30_H_42_O_13_Na, 633.2523).

Excavatoid P (**2**): white powder; mp 154–155 °C; [*α*]_D_^25^ + 14 (*c* 0.05, CHCl_3_); IR (neat) *ν*_max_ 3472, 1784, 1734 cm^−1^; ^13^C NMR (CDCl_3_, 100 MHz) and ^1^H NMR (CDCl_3_, 400 MHz) data, see Table 1; ESIMS *m/z* 685 (M + Na)^+^; HRESIMS *m/z* 685.2235 (Calcd for C_30_H_43_ClO_14_Na, 685.2239).

### 3.4. Human Neutrophil Elastase Release

Human neutrophils were obtained by means of dextran sedimentation and Ficoll centrifugation. Elastase release experiments were performed using MeO-Suc-Ala-Ala-Pro-Valp-nitroanilide as the elastase substrate [8,9].

## Figures and Tables

**Table 1:** ^1^H and ^13^C NMR data for diterpenoids **1** and **2**.

	1		2	
Position	^1^H^a^	^13^C^b^	^1^H^c^	^13^C^d^
1		43.3 (s)^f^		44.0 (s)
2	5.81 d (2.0)^e^	69.3 (d)	4.62 s	88.2 (d)
3	5.13 br s	69.9 (d)	5.07 d (11.6)	68.8 (d)
4	2.25 m (2H)	33.7 (t)	5.82 s	70.9 (d)
5		62.1 (s)		77.8 (s)
6	3.11 d (8.8)	63.0 (d)	4.29 s	65.9 (d)
7	4.69 d (8.8)	78.3 (d)	5.26 s	75.7 (d)
8		72.6 (s)		67.5 (s)
9	5.76 s	68.5 (d)	5.36 d (8.4)	66.0 (d)
10	2.18 br s	45.3 (d)	3.52 dd (8.4, 4.4)	39.5 (d)
11	2.32 br s	34.5 (d)	2.54 m	37.3 (d)
12	3.96 br s	69.3 (d)	4.13 m	66.9 (d)
13	1.92 m (2H)	34.8 (t)	1.83 m (*α*)	30.3 (t)
			1.96 m (*β* )	
14	5.16 br s	73.0 (d)	4.84 br s	80.8 (d)
15	1.52 s	18.2 (q)	0.86 s	18.2 (q)
16	1.35 s	21.1 (q)	1.55 s	22.3 (q)
17		63.3 (s)		60.6 (s)
18	1.57 s	11.1 (q)	1.63 s	10.0 (q)
19		170.8 (s)		170.0 (s)
20	1.19 d (7.2)	16.3 (q)	1.11 d (7.6)	9.0 (q)
OH-3			3.18 d (11.6)	
OH-5			2.36 s	
OH-12	n.o.^g^		2.17 br s	
2-OAc		169.5 (s)		172.0 (s)
	2.12 s	21.1 (q)	2.03 s	21.1 (q)
9-OAc		169.3 (s)		170.4 (s)
	2.18 s	21.2 (q)	2.43 s	21.4 (q)
14-OAc		170.1 (s)		170.3 (s)
	1.96 s	21.1 (q)	2.17 s	21.3 (q)
3-OCOPr		173.6 (s)		
	2.23 m (2H)	35.6 (t)		
	1.64 m (2H)	17.8 (t)		
	0.95 t (7.2)	13.6 (q)		
4-OCOPr				173.9 (s)
			2.33 t (7.6) (2H)	36.3 (t)
			1.66 m (2H)	18.4 (t)
			0.98 t (7.6)	13.7 (q)

a: Spectra were recorded at 400 MHz at 0 °C;

b: Spectra were recorded at 100 MHz at 0 °C;

c: Spectra were recorded at 400 MHz at 25 °C;

d: Spectra were recorded at 100 MHz at 25 °C;

e: *J* values (in Hz) in parentheses;

f: Multiplicity deduced by DEPT and HMQC spectra and indicated by usual symbols;

g: n.o. = not observed.

**Table 2:** The ^1^H-^1^H COSY and HMBC (H→C) correlations for diterpenoids **1** and **2**.

	1		2	
Position	^1^H-^1^H COSY	HMBC	^1^H-^1^H COSY	HMBC
H-2	H-3	C-1, -4, -14, -15, acetate carbonyl	H-3	C-1, -3, -4, -10, -14, acetate carbonyl
H-3	H-2, H_2_-4	C-4	H-2, H-4, OH-3	C-1, -5
H-4	H-3	C-3, -5, -6	H-3	C-2, -5, -16, *n*-butyrate carbonyl
H-6	H-7	n.o.	H-7	C-4, -5, -7, -8, -16
H-7	H-6	C-5, -6, -17, -19	H-6	C-5, -6, -9, -19
H-9	H-10	C-1, -7, -8, -10, -11, acetate carbonyl	H-10	C-7, -8, -10, -11, -17, acetate carbonyl
H-10	H-9, H-11	C-1, -2, -11, -12, -14	H-9, H-11	C-1, -8, -9, -11, -12, -15, -20
H-11	H-10, H-12, H_3_-20	C-10, -20	H-10, H-12, H_3_-20	C-1, -10, -12, -20
H-12	H-11, H_2_-13	n.o.	H-11, H_2_-13	C-20
H-13	H-12, H-14	C-1, -14	H-12, H-14	C-12
H-14	H_2_-13	C-1, -2, -12, -13, acetate carbonyl	H_2_-13	C-10, -12
H-15		C-1, -2, -10, -14		C-1, -2, -10, -14
H-16		C-4, -5, -6		C-4, -5, -6
H-18		C-8, -17, -19		C-8, -17, -19
H-20	H-11	C-10, -11, -12	H-11	C-10, -11, -12
OH-3			H-3	C-3
OH-5				C-4, -5, -16
OH-12	n.o.^a^	n.o.	H-12	C-11

a : n.o. = not observed.
